# Lupus and the Liver: A Case Study

**DOI:** 10.7759/cureus.5477

**Published:** 2019-08-24

**Authors:** Shaan Patel, Michelle Demory Beckler, Marc M Kesselman

**Affiliations:** 1 Miscellaneous, Nova Southeastern University Dr. Kiran C. Patel College of Osteopathic Medicine, Fort Lauderdale, USA; 2 Immunology, Nova Southeastern University Dr. Kiran C. Patel College of Osteopathic Medicine, Fort Lauderdale, USA; 3 Rheumatology, Nova Southeastern University Dr. Kiran C. Patel College of Osteopathic Medicine, Fort Lauderdale, USA

**Keywords:** lupus hepatitis, systemic lupus erythematosus (sle), systemic lupus erythematosus (sle), lupus

## Abstract

Systemic lupus erythematosus (SLE) is an autoimmune disorder with a wide range of systemic manifestations. Though skin, renal, joint, and hematologic involvement are often associated with SLE, hepatitis is not a common manifestation. While clinically significant hepatopathy in SLE is rare, asymptomatic hypertransaminasemia has been seen in up to 60 percent of SLE patients during the course of their disease and is generally attributed to viral hepatitis, hepatotoxic drugs, or alcohol use. A diagnosis of lupus hepatitis is largely considered a diagnosis of exclusion. There has been a correlation between the presence of ribosomal P autoantibodies with the incidence of lupus hepatitis. Generally, lupus hepatitis responds well to therapy with prednisone, although cases refractory to corticosteroids and conventional immunosuppressants have been described. In these cases, treatment with mycophenolate mofetil has been shown to be effective. Here, we present the case of a 15-year old female who presented with a new diagnosis of SLE with an incidental elevation of her liver function tests (LFTs) and a subsequent finding of hepatomegaly with fatty infiltration.

## Introduction

Systemic lupus erythematosus (SLE) is an autoimmune disorder classically associated with malar rash, arthralgias, cytopenias, serositis, renal failure, endocarditis, and antiphospholipid syndrome [1]. However, with the pathophysiology of the disease being based in the production of antinuclear antibodies (ANA) and antibodies against various components of the nucleus, lupus can manifest in nearly any organ system in the body and thus can have a wide range of presenting symptoms [2]. One possible manifestation of SLE is an elevation of liver function tests (LFTs) which, as will be discussed here, can be due to a wide variety of etiologies including the SLE itself [3].

## Case presentation

A 15-year old African American female patient with history of recurrent episodes of eyelid swelling presented to the Emergency Department (ED) with a one-week history of bilateral eyelid swelling and an erythematous rash on her face. She reported an elevated temperature at home with a maximum temperature of 104$^{\circ}$F the day prior to admission, for which she took ibuprofen. Of note, this was her third episode of eyelid swelling in the past year. Previous episodes had resolved with a five-day course of prednisone. However, during this instance while her swelling improved with corticosteroids, her rash presented and worsened leading to the ED visit. The patient had no known allergies, no past medical or surgical history, and was up to date on her immunizations. She also denied any tobacco, alcohol, or illicit substance use and had never been sexually active. On examination, the patient was afebrile, normotensive, with an oxygen saturation of 98 percent on room air and a body mass index (BMI) of 22.9. She was alert, awake, and oriented to person, place, and time. The patient had bilateral eyelid edema, facial edema, an erythematous, nonpruritic, non-tender rash in a malar distribution along the nasolabial folds, cervical and submandibular lymphadenopathy, and oval black discoid erythematous patches along right upper arm. Physical examination was otherwise unremarkable.

**Figure 1:**
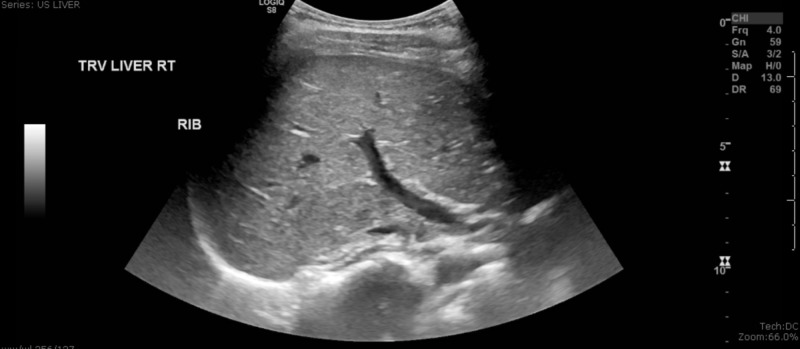
Ultrasound Image of Liver, Right Side Transverse View

Labs were remarkable for an elevated erythrocyte sedimentation rate (ESR) of 46 mm/hr and hemoglobin (Hb) of 11g/dL along with a hypertransaminasemia with aspartate transaminase (AST) and alanine transaminase (ALT) of 154 units/liter and 145 units/liter, respectively (Table 1, 4). Serologies were positive for antinuclear antibodies, anti-Smith, anti-ribonucleoprotein (RNP), anti-chromatin (nucleosomal). She was negative for anti-smooth muscle antibody, anti-mitochondrial, anti-dsDNA, anti-Liver Kidney Microsomal type 1 (LKM1), and antiphospholipid antibody serologies along with a negative hepatitis panel. Monospot, Ebstein-Barr virus (EBV) and cytomegalovirus (CMV) IgM were all negative (Table 2, 3). However, EBV IgG levels were elevated (Table 3). Total IgG and IgE levels were elevated, as well as elevated levels of ferritin, amylase, aldolase, C1 esterase inhibitor with normal levels of C3 and C4 (Table 4). Additionally, acetaminophen, salicylate, and ethanol levels were all low (Table 4). Based on serologies, a diagnosis of SLE was made. An ultrasound evaluation of her liver performed subsequently found fatty infiltration and hepatomegaly (Figure 1). Patient’s symptoms showed improvement once started on methylprednisolone 40mg twice daily and hydroxychloroquine 200mg daily and repeat LFTs showed downtrend with an AST and ALT of 114 units/liter and 137 units/liter, respectively. After discharge from the hospital, she was told to follow-up with the Pediatric Rheumatology outpatient.

**Table 1:** Pertinent Complete Blood Count and Comprehensive Metabolic Panel Values Abbreviations: uL (microliter) dL (deciliter) L (liter) mg (milligram) g (gram) fl (femtoliters) mmol (millimoles)

Lab	Result	Reference Range
White Blood Cells (WBC)	5.23 x 10^3 WBC/uL	4.5-13 x 10^3 WBC/uL
Hemoglobin (Hb)	11.0 g/dL	11.5-15.3 g/dL
Mean Corpuscular Volume (MCV)	86.2 fl	78-100 fl
Platelets (Plt)	172 x 10^3 Plt/uL	140-400 x 10^3 Plt/uL
Blood Urea Nitrogen (BUN)	5 mg/dL	7-19 mg/dL
Creatinine (Cr)	0.8 mg/dL	0.6-1.1 mg/dL
Alkaline Phosphatase (ALP)	71 units/L	40-150 units/L
Aspartate Aminotransferase (AST)	154 units/L	5-34 units/L
Alanine Aminotransferase (ALT)	145 units/L	0-55 units/L

**Table 2:** Positive and Pertinent Negative Serologies mg (milligrams) dl (deciliters) mL (milliliters) IU (international units)

Lab	Result	Reference Range
Antinuclear Antibody (ANA)	Positive	Negative
Anti-Smith	3.3 AI	<1.0 AI
Anti-RNP	6.7 AI	<1.0 AI
Anti-chromatin (nucleosomal)	1.3 AI	<1.0 AI
C1 esterase inhibitor	59 mg/dL	21-39 mg/dL
Anti-Smooth Muscle	Negative	Negative
Anti-Mitochondrial	Negative	Negative
Anti-LKM1	<36 units/mL	<36 units/mL
Anti-dsDNA	<1 IU/mL	<4 IU/mL
Anticardiolipin IgM	<12 mpl	<12 mpl
Anticardiolipin IgG	<14 gpl	<14 gpl
Anti-Beta 2 glycoprotein IgM	<9 SMU	<20 SMU
Anti-Beta 2 glycoprotein IgG	<9 SGU	<20 SGU
Angiotensin Converting Enzyme (ACE)	65 units/L	13-100 units/L

**Table 3:** Viral Serologies mL (milliliters)

Lab	Result	Reference Range
Hepatitis B Surface Antigen (HBsAg)	Non-reactive	Non-reactive
Hepatitis B Core Antibody IgM	Non-reactive	Non-reactive
Anti-Hepatitis A Virus IgM	Non-reactive	Non-reactive
Anti-Hepatitis C Virus IgM	Non-reactive	Non-reactive
EBV Early Antigen D IgG	11.6 units/mL	<9 units/mL
EBV Nuclear Antigen IgG	313 units/mL	<18 units/mL
EBV Viral Capsid Antigen IgG	387 units/mL	<18 units/mL
EBV Viral Capsid Antigen IgM	<36 units/mL	<36 units/mL
Monospot	Negative	Negative
CMV IgM	0.59 units/mL	<0.9 units/mL

**Table 4:** Miscellaneous Labs mm (millimeter) hr (hour) mcg (microgram) ng (nanogram) mg (milligram) mL (milliliter) dL (deciliter) L (liter) kU (kilounits)

Lab	Result	Reference Range
Erythrocyte Sedimentation Rate (ESR)	46 mm/hr	0-10 mm/hr
IgG Level	2318 mg/dL	552-1631 mg/dL
IgE Level	1318 kU/L	<114 kU/L
Ferritin	846 ng/dL	5-204 ng/dL
Amylase	386 units/L	25-125 units/L
C3	126 mg/dL	83-193 mg/dL
C4	22 mg/dL	15-57 mg/dL
Acetaminophen Level	<3 mcg/mL	10-30 mcg/mL
Salicylate Level	<5 mg/dL	15-30 mg/dL
Ethanol Level	<10 mg/dL	<10mg/dL



## Discussion

Based on the underlying etiology, the differential for liver manifestations in a patient with SLE can be categorized as due to non-immunological comorbidities, an overlap of another immunological syndrome, or lupus hepatitis [3]. Liver function test abnormalities have been described during some point of the disease course in up to 60 percent of SLE patients. However, it is usually attributed to non-immunological etiologies such as prior treatment with hepatotoxic drugs, viral hepatitis, or alcohol use [4]. Diagnosing lupus hepatitis as the cause of liver damage is challenging as it may be attributed to another co-existing autoimmune etiology such as Autoimmune Hepatitis (AIH) or Primary Biliary Cirrhosis (PBC) [3]. Another consideration is that AIH, which used to be known as lupoid hepatitis, can present with extrahepatic manifestations such as arthralgias and thus be mistaken for SLE [5]. The liver damage can also be due to the lupus itself, which would be known as lupus hepatitis [3]. Having a schema for classifying the differential of liver damage in a SLE patient is important to not only guide the workup but also in the case of our patient, as diagnosis of lupus hepatitis requires exclusion of all other etiologies.

Bessone, et al. describe lupus hepatitis as a subclinical hepatopathy with asymptomatic elevations in liver enzymes that usually occur in the setting of an active lupus flare [3]. As mentioned, the diagnosis of lupus hepatitis can be difficult due to the large variety of etiologies of liver damage associated with lupus. Further complicating the diagnosis, the liver pathology in lupus hepatitis patients tends to be non-specific [4]. As a result, lupus hepatitis requires viral serologies, autoimmune panels, and other exclusionary testing to be done to rule out other etiologies [3]. As can be expected from the ambiguity in differentiating lupus hepatitis from other etiologies, finding an exact incidence in the literature can be challenging with a large variety in reported incidences [3]. However, one recent study by Zheng, et al., of 504 SLE patients found that the incidence of lupus hepatitis based on the exclusion of all other liver etiologies was 9.3 percent with a higher prevalence in those patients with active SLE [6].

Recent research into liver damage in SLE patients found the presence of different markers that could serve as potential diagnostic markers in the future. The first of these markers and the one that has been most widely studied is the presence of ribosomal P autoantibodies [7]. While positive ribosomal P autoantibody titers are present in up to 30 percent of SLE patients, the presence of positive titers have been found in up to 70 percent of SLE patients with lupus hepatitis [8, 9]. Additionally, this value is significant when compared to the proportion of positive ribosomal P autoantibody titers found in SLE patients with other etiologies of liver damage, such as 17 percent in drug-induced hepatitis and 20 percent in patients with SLE-AIH overlap [9]. Furthermore, positive titers of ribosomal P autoantibody are associated with neuropsychiatric manifestations [10]. It is hypothesized that these autoantibodies are not simply a marker of the disease, but may also be responsible for the underlying pathophysiology of the liver disease by possibly targeting hepatoma cell membrane proteins and upregulating proinflammatory cytokines, ultimately leading to hepatocellular lysis [11, 12]. In one study, immunofluorescence found the presence of epitopes that are antigenically similar to P-proteins on the surface of human hepatoma cells, neuroblastoma cells, and fibroblasts [10]. One other diagnostic modality that may provide a way to differentiate lupus from other liver diseases in the future is the deposit of complement 1q in the liver [6]. Zheng, et al. found that seven out of ten of their lupus hepatitis patients that underwent liver biopsy were positive for intense deposits of complement 1q seen on immunopathology compared to no deposits of complement 1q in patients with other forms of liver disease [6].

Corticosteroids have been found to be an effective treatment modality for lupus hepatitis and a study by Piga, et al. found that the disease generally responds well to moderate to high doses of prednisone with resolution of the disease course [2]. One study by Tagawa, et al. discussed a case of lupus hepatitis that was refractory not only to corticosteroids but also to conventional immunosuppressants such as cyclophosphamide, tacrolimus, and azathioprine [11]. In this case, Tagawa, et al. found that utilizing mycophenolate mofetil led to rapid stabilization and resolution of liver enzyme abnormalities and control of the lupus hepatitis activity despite reduction in methylprednisone dose [11].

## Conclusions

This case demonstrates a unique presentation of SLE with lupus hepatitis. While elevation of LFTs with evidence of hepatic steatosis can be quite common in patients with lupus, this patient is a rare case where one can effectively rule out all other etiologies due to no history of alcohol use, normal BMI, a negative hepatitis panel with negative EBV and CMV IgM, negative anti-smooth muscle, anti-LKM1, and anti-mitochondrial serology, and low levels of acetaminophen, salicylates, and ethanol. Furthermore, the treatment of a patient with a new diagnosis of SLE allows us to rule out the effect of chronic treatment of SLE with hepatotoxic drugs causing the liver damage. While lupus hepatitis is a rare presentation of lupus, it is important to note that it responds well to corticosteroids and in our case LFTs began to downtrend several days after beginning treatment with methylprednisolone.
